# Rapid evolution of a retro-transposable hotspot of ovine genome underlies the alteration of *BMP2* expression and development of fat tails

**DOI:** 10.1186/s12864-019-5620-6

**Published:** 2019-04-02

**Authors:** Zhangyuan Pan, Shengdi Li, Qiuyue Liu, Zhen Wang, Zhengkui Zhou, Ran Di, Xuejiao An, Benpeng Miao, Xiangyu Wang, Wenping Hu, Xiaofei Guo, Shenjin Lv, Fukuan Li, Guohui Ding, Mingxing Chu, Yixue Li

**Affiliations:** 1 Institute of Animal Science, Chinese Academy of Agricultural Sciences, Beijing, China; 2 Key Lab of Computational Biology, CAS-MPG Partner Institute for Computational Biology, Shanghai Institutes for Biological Sciences, Chinese Academy of Sciences, Shanghai, China; 3 College of Agriculture and Forestry Science, Linyi University, Linyi, China; 4 Shanghai Center for Bioinformation Technology, Shanghai Industrial Technology Institute, Shanghai, China

**Keywords:** Retro-transposable hotspot, BMP2, Fat tail, Sheep, Evolution

## Abstract

**Background:**

Sheep have developed the ability to store fat in their tails, which is a unique way of reserving energy to survive a harsh environment. However, the mechanism underlying this adaptive trait remains largely unsolved.

**Results:**

In the present study, we provide evidence for the genetic determinants of fat tails, based on whole genome sequences of 89 individual sheep. A genome-wide scan of selective sweep identified several candidate loci including a region at chromosome 13, a haplotype of which underwent rapid evolution and spread through fat-tailed populations in China and the Middle East. Sequence analysis revealed an inter-genic origin of this locus, which later became a hotspot of ruminant-specific retro-transposon named BovB. Additionally, the candidate locus was validated based on a fat- and thin-tailed cross population. The expression of an upstream gene *BMP2* was differentially regulated between fat-tailed and thin-tailed individuals in tail adipose and several other tissue types.

**Conclusions:**

Our findings suggest the fixation of fat tails in domestic sheep is caused by a selective sweep near a retro-transposable hotspot at chromosome 13, the diversity of which specifically affects the expression of *BMP2*. The present study has shed light onto the understanding of fat metabolism.

**Electronic supplementary material:**

The online version of this article (10.1186/s12864-019-5620-6) contains supplementary material, which is available to authorized users.

## Background

Storing fat is an essential process to temporarily reserve energy, which enables animals to endure harsh environments. Sheep, a major type of livestock, have developed a unique mechanism to store fat in tails, which is present in about 25% of the global sheep population [1]. Fat-tailed sheep are commonly found in Asia and northern Africa [1]. In China, fat-tailed breeds from an ancestral lineage, known as Mongolian sheep, are widely distributed in the northern area [2, 3]. Owing to the fact that the wild ancestors of domestic sheep all have thin tails, it has been suggested that fat tails were developed following domestication as an adaptive response to preserve energy during migration and winter [4, 5]. Fat-tailed sheep are adapted to harsh environments characterized by extreme cold [6], dryness [7], and food scarcity [8]. Excess tail fat serves as a source of energy that improves the probability of survival through the winter [8]. However, over-deposition of fat may compromise fertility and reproductive fitness [9, 10], thus reducing the economic value of the sheep. Consequently, the association between tail fat and reduced fertility and reproductive fitness in sheep has led researchers to investigate the genetic determinants of fat tails. Several studies have provided evidence of crucial genes influencing tail types based on single nucleotide polymorphism (SNP) marker panels [4, 5, 11]. However, a lack of agreement regarding the affected loci between different fat-tailed populations suggests a complicated mechanism underlying this adaptive trait. Moreover, the application of marker panels is efficient in searching for regions with signals of selection, but often has a restricted power to detect the causative genes or variants.

In the present study, we provide evidence of a selective sweep in Chinese fat-tailed sheep based on whole-genome sequencing of 49 Mongolian (fat-tailed), 30 Tibetan (thin-tailed) and 10 European (thin-tailed) sheep. We evaluated the signatures of positive selection, including a top signal at chromosome 13 identified by independent studies but still with conflicting understandings of causative genes [4, 11]. We studied the sequence origin of this locus by inter-species comparisons and analyzed gene expression patterns in different tail types. The aim of this study was to understand the lineage evolution of domestic sheep and clarify the potential mechanism underlying their essential phenotypes in adaptation.

## Results

### Analysis of a selective sweep based on whole-genome sequencing of domestic sheep

**Fig. 1:**
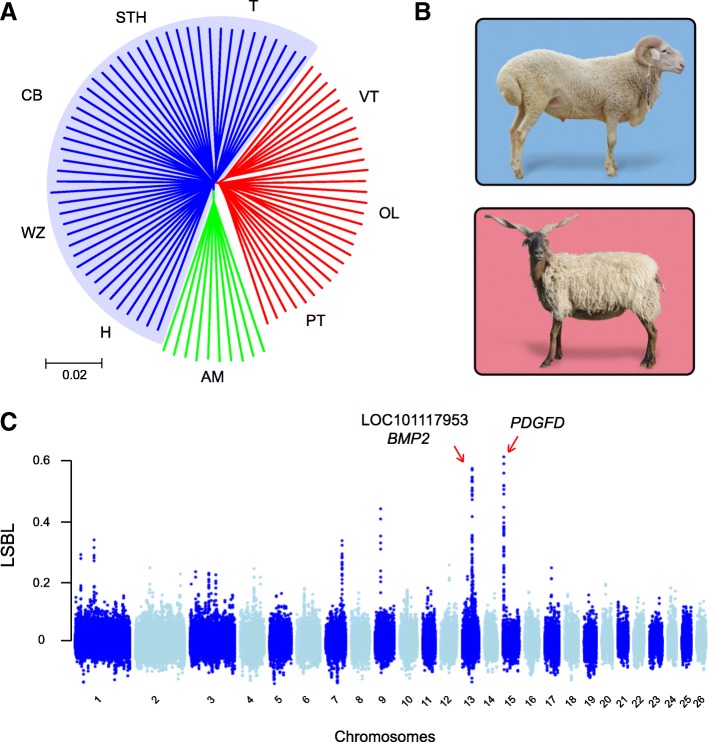
Selective sweeps in Chinese fat-tailed sheep. **a** Phylogenetic structure of Chinese Mongolian breeds (fat-tailed; clade in blue), Chinese Tibetan breeds (thin-tailed; clade in red) and a European-origin breed (thin-tailed; clade in green). T, Tan; STH, Small Tail Han; CB, Cele Black; WZ, Wuzhumuqin; H, Hu; VT, Valley Tibetan; PT, Prairie Tibetan; OL, Oula; AM, Australian Merino. **b** Phenotypic features of individuals from PT breed (top) and STH breed (bottom). **c** Manhattan plot of selective sweep statistic *LSBL* over 26 ovine chromosomes

To elevate our understanding of ovine lineage evolution, we analyzed the abundant genetic variants (~ 40 million) generated from whole-genome sequencing (WGS) data of 89 sheep (Materials and Methods). The genetic relationship between these individuals has been well described elsewhere [12], suggesting that 49 out of the 89 samples from five Chinese Mongolian sheep breeds (MGS) form a major ovine lineage in China (the genetic distances between these breeds are small), distinct from the Chinese Tibetan sheep breeds (TBS) and European sheep breeds (EUS). The phylogenetic structure of these lineages (Fig. 1a) is consistent with the mito-genomic evidence of ovine migrating trajectory from Eurasia to China [13].

We next calculated the lineage-specific branch length (*LSBL*) [14, 15] of MGS on a sliding-window basis (Materials and Methods). The statistic measures the extent of population differentiation in the MGS clade by comparing them with TBS and EUS, and is therefore a powerful indicator of a recent selective sweep. As displayed in the Manhattan plot (Fig. 1c), two highly significant signals of high *LSBL* values (*LSBL*_top_ = 0.574 and *LSBL*_top_ = 0.613) were found in chromosome 13 and 15. Both signals were supported by extensive numbers of high-*LSBL* windows (Additional file 1: Table S1) because of a hitchhiking effect on the variants in linkage with the selected sites. In particular, a sweep signal at chromosome 13 was consistent with the results from independent studies of fat-tailed populations [4, 11], nevertheless its causative event is controversial because the region contains multiple genes. Specifically, Moioli et al. [4] suggested the bone morphogenetic protein 2 (*BMP2*), while Wei et al. [11] suggested the protein phosphatase 1 catalytic subunit gamma (*PPP1CC*) to be causative (it is a paralogue of the original *PPP1CC* gene at ovine chromosome 13 with NCBI gene identifier LOC101117953).

### Genomic region encompassing LOC101117953 underwent a selective sweep in Mongolian sheep

**Fig. 2:**
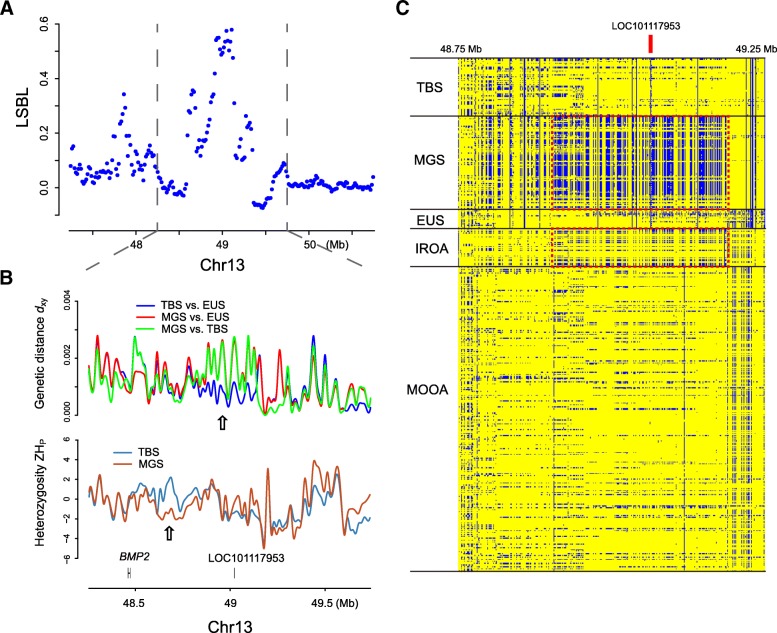
Evidence of selection in fat-tailed breeds at ovine chromosome 13. **a** A local plot of *LSBL* at ovine chromosome 13. **b** Other evidences of selection including pair-wise genetic differences *d*_xy_ between lineages (TBS, Tibetan sheep; MGS, Mongolian sheep; EUS, European sheep), as well as intra-lineage heterozygosity *H*_P_. **c** A variant heatmap shows haplotypic structures of 89 sheep from our WGS dataset (MGS, TBS, EUS), 20 sheep from Iran (IROA) and 160 sheep from Morocco (MOOA). Colors denotes different genotypes at each bi-allelic variant site

Due to the genetic hitchhiking among variants in close proximity, it is often difficult to assess the true causative mutations underlying the alterations of phenotype and fitness. As WGS of ovine population provided a novel opportunity to study their genomic architectures at higher resolution, we further analyzed the inter- and intra-population diversities of the highly significant sweep regions (Fig. 2 and Additional file 2: Figure S1). An obvious peak point of *LSBL* values was observed at position ~ 49 M bases (Mb) on chromosome 13 (Fig. 2a). As expected, the signal strength was attenuated by increasing distance to the peak-point, and became insignificant after ~ 0.5 Mb extension on either side. Two protein-coding genes were annotated in this region: a newly characterized gene LOC101117953 is in the center of high-*LSBL* block, while *BMP2* is upstream to it near the border (Fig. 2b). A region encompassing LOC101117953 of ~ 250 kb exhibited excessive population differentiation (measured by genetic distance *d*_xy_) between MGS vs. EUS and MGS vs. TBS, but not TBS vs. EUS (Fig. 2b). Moreover, the region also showed a depletion of intra-population diversities (measured by heterozygosity *ZH*_P_) in MGS (Fig. 2b). These findings collectively indicate that the Mongolian lineage has experienced a recent sweep over the locus encompassing LOC101117953.

We then questioned whether this locus could explain fat-tailed phenotypes in ovine populations outside of China. By comparing the variant distributions among sheep from China, Iran (IROA) and northern Africa (MOOA), we noticed a haplotype sweeping through the MGS population was also present in IROA at high frequency (Fig. 2c), which is in agreement with the fact that many Iranian breeds have fat tails [1]. Although it is unknown whether the locus is also under selection in other fat-tailed populations, the data here suggested the alternative haplotype has been derived in domestic sheep before their spread in China.

### LOC101117953 is derived from retro-transposable event

**Fig. 3:**
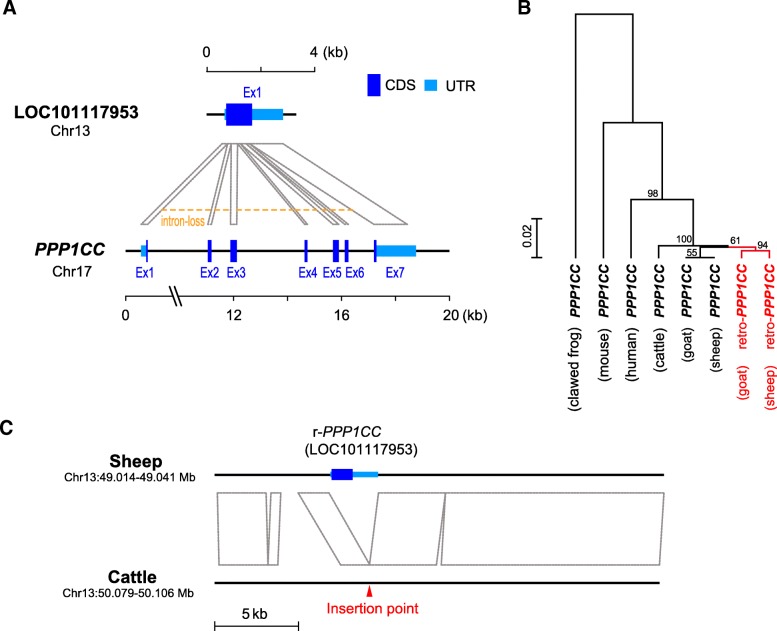
Retro-transposable origin of gene LOC101117953. **a** Alignment between ovine sequences of LOC101117953 and *PPP1CC*. **b** Gene tree constructed based on sequences of *PPP1CC* and its retro-paralogs (r-*PPP1CC*) from different species. **c** Genomic alignment between sheep and cattle shows the insertion point of r-*PPP1CC*

As a newly characterized gene, LOC101117953 is annotated as a paralogue of *PPP1CC* based on sequence similarity. Although dosage effect (assuming LOC101117953 has a function similar to that of *PPP1CC*) is a possible mechanism underlying how LOC101117953 may yield a fitness advantage to the fat-tailed ovine lineage, it is not completely convincing due to a lack of knowledge about this novel gene. To consider more possibilities, we performed analysis to categorize and date the origin of LOC101117953, which is essential for understanding its functionality. Intriguingly, the alignment of two paralogue gene sequences (with introns) suggested a retro-transposable origin of LOC101117953 from *PPP1CC*, evidenced by clear intron losses in the derived locus (Fig. 3a). Moreover, as loss of introns can be attributed to either retro-transposition or artifacts from RNA contamination in the sequencing library, primers were designed to capture and validate the DNA sequence of LOC101117953 based on the classical Sanger sequencing method [16, 17]. This experiment confirmed the existence of gene duplication (Additional file 2: Figure S2A). In addition, there is an artificial intron of LOC101117953 (homologous to the 4th intron of *PPP1CC*) in the reference genome compared with ensuing validation (Additional file 2: Figure S2B), which is a likely assembly error due to the limitation of short-read sequencing.

To date the origin of LOC101117953, which has now been proven as the retro-copy of *PPP1CC* (r-*PPP1CC*), nucleotide BLAST [18] was performed against six vertebrate genomes making the ovine r-*PPP1CC* sequence as the query. The orthologues were determined by both sequence similarity and shared synteny among species: true r-*PPP1CC* orthologues were located at the intergenic region between *BMP2* and *HAO1* (IBH region). Among all the searched vertebrates, orthologs of r-*PPP1CC* were merely found in two *Caprinae* species: sheep and goat (sub-family *Caprinae* is inside ruminant family *Bovidae*). By sequence alignment of this region between sheep and cattle, the insertion point of the retro-transposon was clearly observed (Fig. 3b), suggesting the exact time of the retro-transposable event after the coalescence of family *Bovidae* (~ 26.5 Ma, estimated by TimeTree [19]) and before that of sub-family *Caprinae* (~ 12.5 Ma). Moreover, the phylogenetic structure of all *PPP1CC* homologues confirmed the same emergence time of r-*PPP1CC* within the sub-clade comprising sheep and goat (Fig. 3c). Taken together, these findings demonstrate that LOC101117953 (r-*PPP1CC*) is a novel gene copy derived from a retro-transposable event, which has been present in part of the ruminant family.

### IBH region of sheep is a ruminant-specific retro-transposable hotspot

**Fig. 4:**
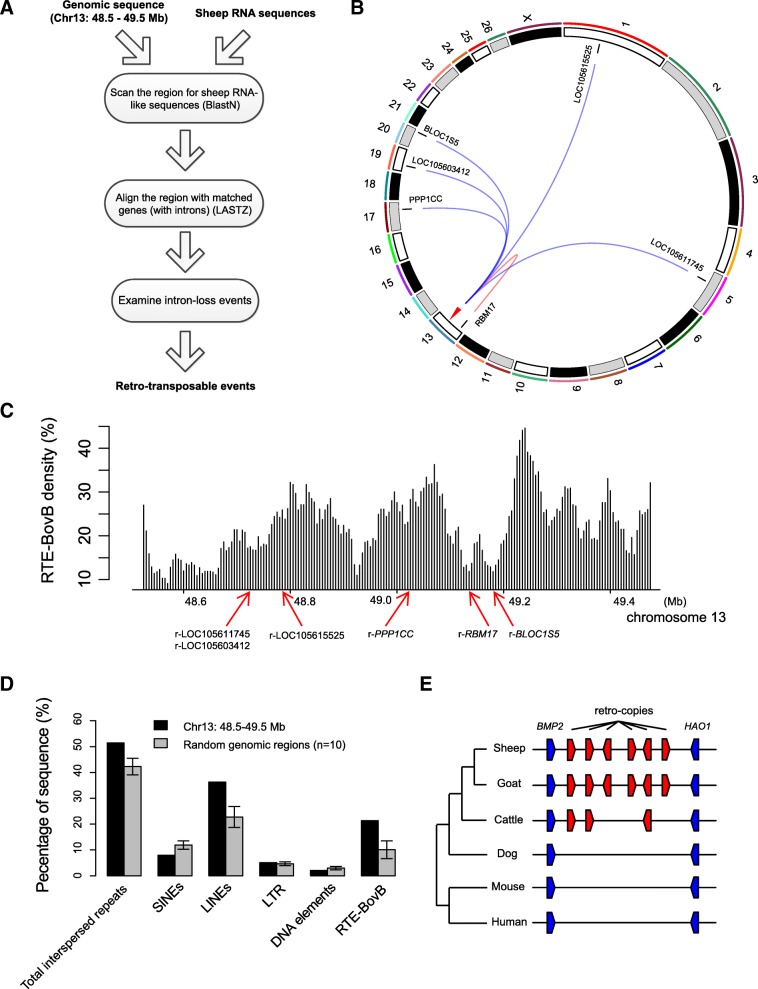
Identification of retro-transposable events at IBH region. **a** Scheme for identifying retro-transposable events at IBH region, specifically from 48.5 to 49.5 Mb at chromosome 13. **b** A circular plot of ovine chromosomes and six retro-transposable events detected at IBH region. **c** Sliding-window-based densities of the RTE BovB, calculated from the prediction of functional repeats by RepeatMasker. **d** Comparison of RTE occupation between IBH and random genomic regions (*n* = 10). Error bars denotes standard deviations. **e** The positions and emergence time of six retro-copies estimated based on a cross-species comparison

Although a single retro-transposable event can occur in isolation, retro-transposons derived from processed RNA are often clustered at common loci of the genome [20]. Thus, we questioned whether r-*PPP1CC* was the only retro-copy of actively transcribing genes in the IBH region. To test this, the genomic sequence from 48.5 Mb to 49.5 Mb of chromosome 13 was compared with the ovine RNA database to search for potential RNA-derived sequences, and afterwards these sequences were aligned with their normal paralogues to confirm the absence of introns (Fig. 4a). In total, eight candidate RNA-derived sequences were identified, in which six were confirmed as retro-copies with clear evidence of intron deletion. (Additional file 2: Figure S3). Among the six retro-copies, five were from different chromosomes (r-*PPP1CC*, r-*BLOC1S5*, r-LOC105603412, r-LOC105615525, r-LOC105611745), and one was from a distant location of chromosome 13 (r-*RBM17*) (Fig. 4b). Given these, the IBH region of sheep seems to be a hotspot of retro-transposable events, which comprises six or even more undetected gene copies derived from intron-less RNA.

There are various types of retro-transposable elements (RTE) in mammals, including long terminal repeat (LTR) and non-LTR retro-transposons [21]. We now have proven that at least six retro-copies of processed transcripts were clustered at the IBH region of ovine genome, and it is therefore intriguing to know which type of RTEs have contributed to anchoring these retro-transposons. By annotating the 1 Mb IBH region with known interspersed repeats of mammal genomes (Table 1), an impressively high proportion of the sequence (21.32%) was identified as a ruminant-specific long interspersed nuclear element (LINE) named BovB [22]. Density of this repeat sequence ranged from about 10 to 45% when scanning the whole region with a 50-kb sliding window (Fig. 4c). In addition to this, L1 repeats known as the dominant retro-transposon type in mammals [23] comprised 14% of the locus, while other types including short (S) INEs and LTR elements comprised 7.95 and 5.07% (Table 1).

**Table 1:** Transposable elements detected in 1 Mb IBH region

Element class	Subclass	Most frequent elements	Number of elements	Percentage on region
SINEs	MIRs	MIR, MAR1	134	1.26%
	tRNA & core-RTE	Bov-A2, Bov-tA	518	6.69%
LINEs	L1	L1	248	14%
	L2	L2	48	1.18%
	CR1(L3)	L3	7	0.11%
	RTE-BovB	BovB	304	21.32%
LTR elements	ERVL	ERVL, LTR16	32	1.99%
	ERVL-MaLRs	MLT1	36	0.91%
	ERV classI	ERV1, ERV54, LTR1	29	1.19%
	ERV classII	BTLTR1	75	0.89%
DNA elements		MERs, hAT-Charlie, TcMars-Tigger	98	2.09%

To test whether IBH region is a favorable location of RTE insertion, we compared the density of each retro-transposon type between IBH vs. random loci (*n* = 10). Impressively, LINEs comprised an obviously higher proportion in IBH region than in random locations (IBH: 36.29%; random: 22.76 ± 4.09%), due to a profound enrichment of BovB repeats (IBH: 21.32%; random: 10.07 ± 3.45%). By contrast, other transposon types (SINEs, LTRs, DNA elements) showed either less or equal occupations of IBH compared to random locations (Fig. 4d).

Given these, BovB seems to be the most likely RTE type which has mediated frequent retro-transposon insertions into IBH region comprising over six retro-copies of gene transcripts. Moreover, this specificity is further evidenced by a consistent chronological order between the initial emergence of RTE and the subsequent retro-transpositions. BovB was proposed to have been horizontally transferred from squamata to ruminants [22, 24], therefore BovB-related retro-transposable events should not precede the coalescent time of ruminant family and the retro-copies should be present merely in ruminant species. This is exactly the case for the retro-transposons identified in IBH, where sheep and goat carry all six retro-copies, cattle carry three of them, and none is present in other vertebrate species (Fig. 4e).

### Haplotype of the IBH region predicts tail phenotypes in a hybrid population

Though we have corroborated that tail types of Chinese sheep are highly consistent with their IBH genotypes, it is possible that the selective sweep at the IBH region may affect other phenotypes distinguished between fat-tailed and thin-tailed populations. This is often the case when the studied phenotype is correlated with population structures, which means the genetic differentiation between groups may have various explanations. To fully address this question, we tested whether IBH haplotype could predict tail phenotypes within a hybrid population with diverse shapes of tail (Additional file 2: Figure S4). Due to chromosomal recombination, only the association between the causative locus and tail phenotypes will be steadily passed on over generations, thus enabling the exclusion of loci affecting other phenotypic outcomes.

**Fig. 5:**
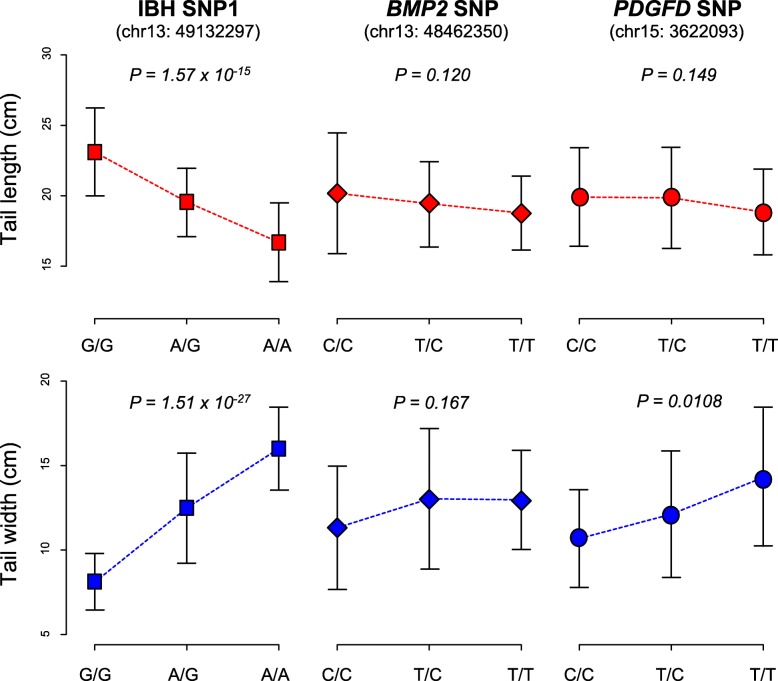
Predictive power of candidate SNPs on tail phenotypes. Strength of association was tested between candidate SNPs (IBH SNP1, IBH SNP2, *BMP2* SNP and *PDGFD* SNP) and tail phenotypes (length and width). Two IBH markers are in complete LD, therefore only one is showed. *P* values were calculated based on linear regressions after adjustment for age and sex (assuming an additive effect of single allele). A more complete analysis is in Additional file 1: Table S4

The hybrid population we used comprises 116 individuals (28 females and 88 males), in which four genetic markers were genotyped (two for IBH region, one for *BMP2* and one for *PDGFD*, see Additional file 1: Table S2 and S3). Tail length and width, which are known to correlate with fat storage capability, were measured for all individuals. Linear regressions were performed to evaluate the association between genetic markers and tail phenotypes, based on adjustment of age and gender (Additional file 1: Table S4). Intriguingly, two IBH markers (which are in complete LD) showed dramatically strong association with tail length and width (Fig. 5). The trend best fits an additive genetic model, where each mutated allele accumulatively confers ~ 3.3 cm length decrease (*P*-value = 1.57 × 10^− 15^) and ~ 3.5 cm width increase (P-value = 1.51 × 10^− 27^). With this “smoking gun”, we demonstrated that the IBH genotype in Mongolian sheep confers their short and fat tails.

The genotype of a *BMP2* missense variant was slightly correlated with tail width without considering covariates (additive model: P-value = 0.058; dominant model: P-value = 0.025), nevertheless the association became insignificant after adjustment for age and gender (additive model: P-value = 0.167; dominant model: P-value = 0.245). *PDGFD* marker shows a moderate correlation with tail width after adjustment (additive model: P-value = 0.0108), whilst it seems not to affect tail length (additive model: P-value = 0.149). This result suggests *PDGFD* does not has a strong effect on tail phenotypes: although it shows the highest *LSBL* value in fat-tailed sheep (Fig. 1c), its correlation with tail types is poorly detected in hybrid populations because random chromosomal recombination “diluted” the linkage between distant loci.

### Inference of the causative gene by gene expression profiles

Given that LOC101117953 is a retro-copy of *PPP1CC*, we assume it is less likely to be the causative gene for tail phenotypes, although its gene body is located with the *LSBL* peak. The mechanism of retro-transposition means that most retro-copies are pseudo-genes, because the promoter regions are often not transcribed and therefore not transposed with RTEs [20]. To test whether LOC101117953 is a pseudo-gene, we performed quantitative PCR (qPCR) in 17 tissues of thin-tailed and fat-tailed sheep (Materials and Methods). Expression of LOC101117953 was not detected (Additional file 2: Figure S5). Moreover, the putative protein sequence of LOC101117953 was truncated into 188 amino acids (AA) by a novel stop codon compared with the 323 AA protein of *PPP1CC* (Additional file 2: Figure S6). A neutral test based on non-synonymous vs. synonymous mutation rate ratio (*ω*) for the *PPP1CC* gene tree (Fig. 4b) revealed a much stronger trend of purification selection in the clade of *PPP1CC* (*ω* = 0.00562) than r-*PPP1CC* (*ω* = 0.1811). Additionally, no statistical significance (*P* = 0.154) was detected to reject neutral evolution of r-*PPP1CC* clade (Materials and Methods, Additional file 1: Table S6). These results suggested that LOC101117953 is not likely to possess essential functions, either at transcript or protein level.

**Fig. 6:**
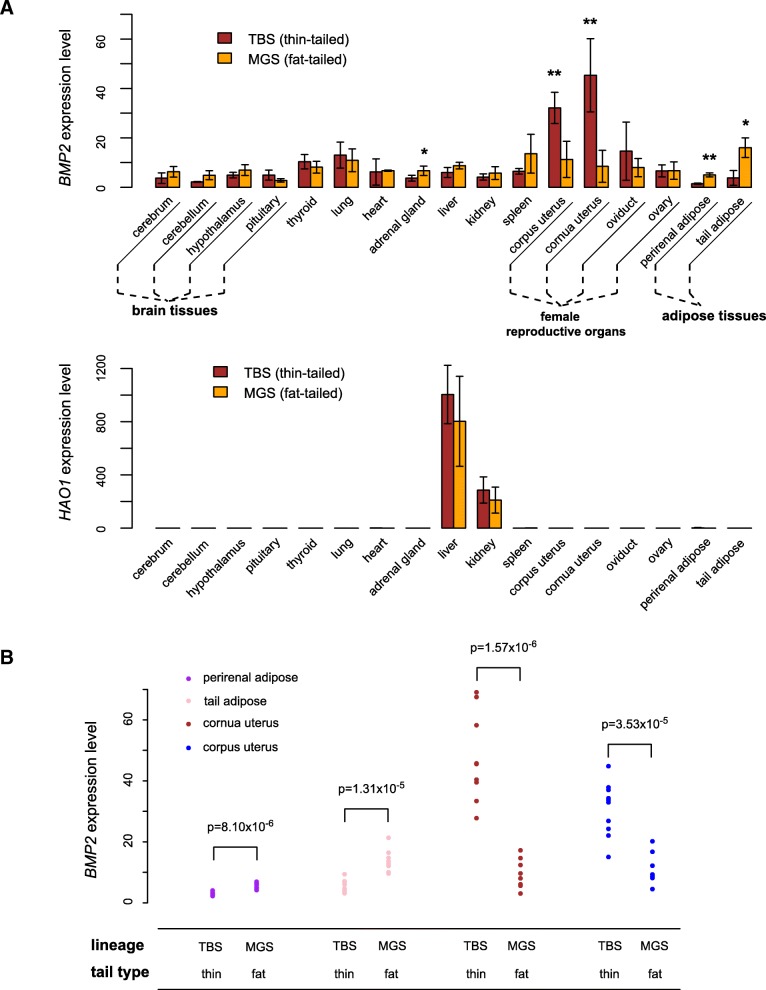
Gene expression difference between fat-tailed and thin-tailed sheep. **a** Comparison of gene expression level for *BMP2* and *HAO1* in 17 tissue types between TBS and MGS (*n* = 4 for each). **, student’s t test P value < 0.01; *, student’s t test P value < 0.05. **b** Validation of *BMP2* expression in adipose and uterine tissues using additional samples (*n* = 8 for each)

We next questioned whether two protein-coding genes *BMP2* and *HAO1*, based on which the borders of IBH were defined, were relevant to tail phenotypes. Although the variant under selection is unlikely to be a protein-altering or intronic mutation within their gene bodies (because they are too distant to the sweep region), we tested the hypothesis whether *BMP2* and *HAO1* gene expression was altered because of the sweep. Surprisingly, *BMP2* but not *HAO1*, showed different tissue expression profiles between fat-tailed and thin-tailed groups (Fig. 6). By quantitative PCR, *BMP2* expression differences were detected in 5 out of the 17 tissue types, in a condition-dependent manner: The fat tailed group is correlated with enhanced *BMP2* transcript levels in perirenal adipose, tail adipose and adrenal gland, but with suppressed expressions in corpus and cornua uterus (Fig. 6a). We repeated the experiments in adipose and uterine tissues with additional biological replicates (*n* = 8 for each tail type), the result of which corroborated our finding and was supported by statistical significance (Fig. 6b). *BMP2* is known to induce both osteoblastic and adipocytic differentiation of pluripotent stem cells [25–28], thus it seems rational that fat-tailed sheep exhibit a ~ 3-fold higher *BMP2* expression in their tail adipose tissues, consistent with a better capability of fat storage. On a different note, *BMP2* also plays an important role in uterine decidualization [29–31], therefore the expression pattern of *BMP2* suggests that fail-tailed phenotype might be accompanied with alterations in uterine functions, which remains to be investigated.

## Discussion

Various animal models have been developed to understand fat metabolism and the related disorders in humans. Abnormal body fat distribution is a common risk factor of various prevalent diseases [32–36]. Sheep, as a world-widely distributed livestock, have developed phenotypic variations in fat storage ability during their domestication, and therefore possess great potential to become a new model organism for studying fat deposition mechanically. Understanding the natural machinery in the sheep tail, which has evolved to accommodate massive deposits of lipid, could be the key to resolve how fat distribution is regulated across tissues.

Positive selection is a critical driving force for organisms to evolve new functions. A handful of methods have been developed to detect positive selection by measuring a selective sweep of genetic diversities [37]. In most applications, the aim is to determine the causative variants (or genes) that affect fitness. However, this is challenged by numerous confounding factors (e.g. genetic hitchhiking, population admixture) in practice. Here we showed that in sheep a selective sweep at the IBH region of chromosome 13, which has been suggested as the determinant of fat deposition and tail type differences [4, 11], is more complex than previously expected. Based on WGS of domestic sheep, we revisited the regions under selective sweep in Mongolian ovine populations. We provided novel evidence to show that the IBH region encompassing LOC101117953 has experienced a recent sweep in fat-tailed sheep. Moreover, data from a hybrid ovine population, for the first time, corroborated the hypothesis that the IBH genotypes confer tail differences in sheep.

**Fig. 7:**
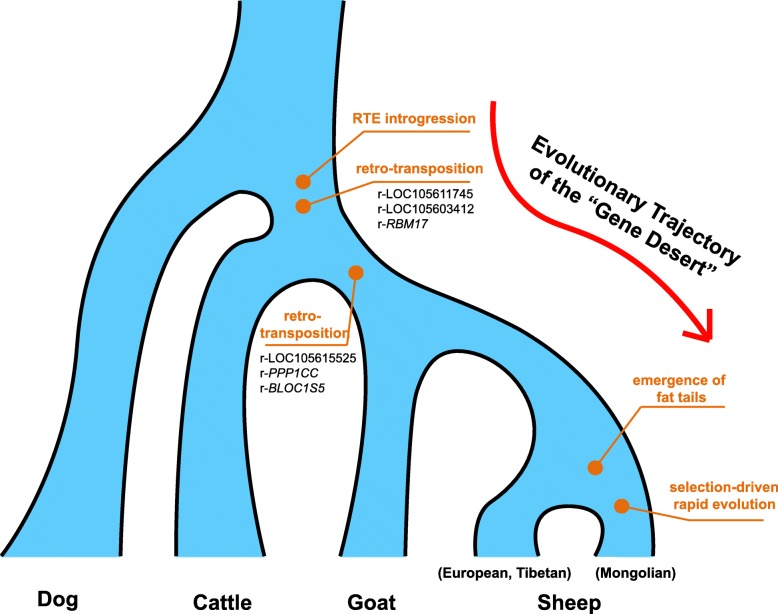
Evolutionary trajectory of IBH region as a target of recent selection in fat-tailed sheep. The river of life summarizes from upstream to downstream the timings of several key events, which contributed to the evolution of IBH sequence, as well as its recent sweep in fat-tailed sheep

Retro-transposable elements comprise a large fraction of the eukaryotic genomes. These genomic elements are considered to be the “seeds of evolution”, because they have played an important role in molecular evolution [38]. Here our data analyses show that the IBH region serves as a hotspot of BovB-related retro-transpositions, which has gradually accumulated after the first introgression of BovB elements into the genome of ruminant ancestor (Fig. 7). These results also corroborate the fact that ruminant-specific RTEs are essential to the generation of novel gene copies on sheep genome. Moreover, a higher insertion frequency of species-specific RTEs compared with random loci suggests a role in species-specific functions [39]. Although the structural alterations introduced by active retro-transposons are usually more deleterious than single nucleotide substitutions, their frequent occurrences in hotspots provide more variations to be favored by natural selection. Since the mutation rate is relatively constant within a species, enrichment of retro-transposable variations potentially enables the species to rapidly evolve novel functions within a short time.

Another key question our study has addressed is the mechanism by which the selective sweep near LOC101117953 has affected fat deposition in sheep tails. Most retro-copies are considered non-functional (pseudo-genes), which significantly contribute to the genomic evolution but are not important for survival [39], so it is surprising that the IBH locus has received intense positive selection during the evolution of fat-tailed sheep. Although no evidence of transcription or potential protein function of LOC101117953 was found, our finding suggests the distant *BMP2* gene is differentially expressed between fat-tailed and thin-tailed sheep in many tissues, including tail adipose. Thus, we hypothesize that the high expression of *BMP2* in tails is likely to be the cause of tail fat deposition in sheep, which is well supported by the function of *BMP2* to induce commitment of stem cells into adipocytes [25–28]. A recent report also showed that fat tails are correlated with increasing number and size of adipocytes [40], where *BMP2* could play a role. Intriguingly, human HiC data (ESC cell) suggests that BMP2 interacts frequently with its downstream regions compatible with a similar location as LOC101117953 in sheep. However, it’s possible that chromatin architecture changes in sheep as consequence of the BovB retron accumulation, therefore more studies using sheep data are necessary for clarifying the role of LOC101117953 in the differential expression of *BMP2*.

Surprisingly, *BMP2* is significantly down-regulated in the uterus of fat-tailed females. This unexpected finding raises the question of whether the advantageous variants for fat reservation in sheep are directly associated with their reproductive traits. On the surface, this is seemingly the case, because many fat-tailed lineages (CB, H, STH) are well known for their fertility [41]. Barbarine sheep, a breed in Tunisia, is well adapted to an environment characterized by seasonally fluctuating availability of feed resources, mainly because of its high fertility and fat tail. *BMP2* was also involved in mouse decidualization, a process required for implantation of the embryo and is driven by estrogen and progesterone receptors [42]. Furthermore, one report showed that *BMP2* enhanced oestradiol production in granulosa cells cultured in vitro [43]. Hence, *BMP2* might play a role in the estrous cycle of sheep to affect fertility by regulating oestradiol production, though the BMP2 expression levels in the reproductive tract have not yet been determined. However, to fully address this question, future work is required including a comprehensive measurement of reproductive traits in various populations.

## Conclusion

In summary, our data suggest that the ovine genome has encountered a recent selective sweep at the IBH locus, which confer the ability of reserving fat in tails. Comparative analysis reveals the IBH locus as a ruminant-specific retro-transposable hotspot, which plays an important role in ovine lineage evolution. Gene expression quantification indicates that the retro-gene near the sweep is poorly functional, but the locus potentially serves as a regulatory element that shifted the expression of distant *BMP2* gene. The present study has filled a gap in the understanding of adaptive evolution of sheep tails and highlights the importance of species-specific RTE in advancing the emergence of species-specific adaptive traits. In addition, our results provide new insight into fat metabolism and novel opportunities for developing therapies for complex metabolic diseases.

## Methods

### Variant calling from whole-genome sequencing data

Genomic variants of 89 sheep were extracted from our previous collection of sheep whole-genome sequences [12, 44], of which 10 individuals from Bayinbuluke breed were not used because of population admixture. Raw read processing and mapping were performed as previously described [12]. An updated variant calling protocol was applied to detect single nucleotide substitutions and short insertions/deletions [45]: 1) use GATK [46] for base quality recalibrations, insertion/deletion realignment and variant discovery; 2) genotyping was performed across all 89 samples simultaneously. Finally, BEAGLE v4.1 [47] was used to impute missing genotypes using parameter “-gtgl”.

Variant files for Iranian (*n* = 20) and Moroccan sheep (*n* = 160) in vcf format were downloaded from the NextGen project webpage (http://projects.ensembl.org/nextgen/).

### Phylogenetic tree construction

The variant data was pruned by PLINK v1.07 [48] with parameter “--indep-pairwise 50 5 0.2” in order to generate a subset of SNPs. Pruned variants were then used to calculate the identity-by-state (IBS) distance matrix of 89 individuals using the “--genome” option. Phylogenetic tree was constructed by PHYLIP [49], using the Neighbor Joining (NJ) algorithm.

### Selective sweep analysis

The lineage-specific branch length (*LSBL*) statistic was used to search for regions that underwent a selective sweep in MGS. Calculation of *LSBL* in MGS (*LSBL*_*M*_) based on a 30-kb sliding window with a 15-kb step was performed by our in-house perl script, which can be broken down into two steps: (1) calculate the fixation index *FI* (or *F*_ST_) between MGS vs. TBS, MGS vs. EUS and TBS vs. EUS, which are represented by *FI*_*M, T*_, *FI*_*M, E*_ and *FI*_*T, E*_; (2) calculate *LSBL*_*M*_ using formula:

1 $$ {LSBL}_M=\frac{FI_{M,T}+{FI}_{M,E}-{FI}_{T,E}}{2} $$

Next, we used two window-based statistics: (1) pairwise nucleotide differences *d*_xy_; and (2) heterozygosity *ZH*_P_ to visualize the selective sweeps at chromosome 13 and 15. Given two populations *x* and *y*, and a genomic region with *n* variants, we first calculated in each population the major and minor allele frequencies of the *k*th variant *f*_*k*, *major*_ and *f*_*k*, *minor*_. Then, *d*_*xy*_ was calculated as:

2 $$ {d}_{xy}=\sum \limits_{k=1}^n{f}_{k,x, major}\sum \limits_{k=1}^n{f}_{k,y, minor}+\sum \limits_{k=1}^n{f}_{k,y, major}\sum \limits_{k=1}^n{f}_{k,x, minor} $$

And the intra-population heterozygosity *H*_P_ was calculated as:

3 $$ {H}_{\mathrm{P}}=\frac{2\cdot \sum \limits_{k=1}^n{f}_{k, major}\sum \limits_{k=1}^n{f}_{k, minor}}{{\left(\sum \limits_{k=1}^n{f}_{k, major}+\sum \limits_{k=1}^n{f}_{k, minor}\right)}^2} $$

Subsequently, *H*_P_ was transformed into the standardized *ZH*_P_:

4 $$ {ZH}_{\mathrm{P}}=\frac{H_{\mathrm{P}}-\mu }{\sigma } $$

In the formula, *μ* denotes the mean and *σ* denotes the standard deviation of *H*_P_.

### Experimental validation of LOC101117953 gene sequence

Two pairs of primers (Additional file 1: Table S5) were designed for cloning the complete DNA sequence of LOC101117953. The primers targeted two overlapped fragments of 1487 bp and 2978 bp as displayed on the electrophoresis gel (Additional file 2: Figure S2A), which were subsequently assembled into a 3458-bp complete sequence and were compared with the reference genome (Additional file 2: Figure S2B).

### Identification of retro-transposable events and retro-transposable element analysis

The process of identifying retro-transposable events was described in Fig. 4a. First, BLASTN search, setting the 1 Mb IBH sequence as query, was performed against the reference RNA sequences of sheep (https://blast.ncbi.nlm.nih.gov/Blast.cgi? PROGRAM = blastn&PAGE_TYPE = BlastSearch&LINK_LOC = blasthome; database set as “Reference RNA sequences” and organism set as “sheep”). RNA sequences with > 500 bp aligned to IBH and > 80% identity were chosen as candidate duplication events. Next, their complete gene sequences (with introns) were extracted and re-aligned with IBH sequence using LASTZ v1.04.00 [50]. Finally, we used dot plots to visualize their alignments with IBH region to confirm the loss of intron sequences (Additional file 2: Figure S3).

RepeatMasker (http://www.repeatmasker.org) was used to annotate RTEs on the IBH sequence, with DNA source chosen as “mammal”.

### Genotype-phenotype association study

Venous jugular blood samples were collected from 116 sheep from a crossbreed (about 4–6 generations) between STH sheep (fat-tailed) and Dorper sheep (thin-tailed) in Lan Ling Shun Yuan Farm of Shandong province. Whole blood was used for DNA extraction by phenol–chloroform method. SNP markers which represent MGS haplotypes were chosen based on their single-nucleotide *LSBL* value: a high value suggests MGS carries distinct genotypes at the site compared with TBS and EUS. Four SNPs representing three genomic regions (IBH, *BMP2* and *PDGFD*) were genotyped in all 116 samples (Additional file 1: Table S2 and S3).

Polymerase chain reactions (PCR) were performed in volumes of 20 μL including 10 μL *Taq* PCR MasterMix (TIANGEN, Beijing), 0.5 μL each of forward and reverse primer, 1 μL DNA, and additional ddH_2_O. The program was run on a Mastercycler 5333 (Eppendorf AG, Hamburg, Germany) according to the following procedure: 5 min at 95 °C for initial denaturation, 32 cycles of 30 s each at 95 °C, 30 s at the appropriate temperature as shown in Additional file 1: Table S5, 30 s at 72 °C, and final extension for 8 min at 72 °C. The PCR products were validated by electrophoresis on 1.5% agarose gels (Promega, Madison, WI, USA) and sequenced by Sangon Biotech (Shanghai) Co., Ltd.

Association test between tail phenotypes (tail length and width) and individual genotypes were performed using linear regressions (with our in-house R script) (Additional file 1: Table S4). Three different genetic models (dominant, recessive and additive effect of single mutated allele) were considered and tested separately. To test confounding effects, individual age and sex was included in regression models as covariates.

### Gene expression quantification of potentially causative genes

Expression profiles of four genes (*HAO1*, *BMP2*, LOC101117953 and *PPP1CC*) in 17 tissue types of TBS and MGS were first examined by RT-PCR (Additional file 2: Figure S5). Primer sequences were shown in Additional file 1: Table S5. Samples from TBS and MGS originated from the Dangxiong farm of Tibetan province and Small Tail Han sheep breeding farm of Shandong province, respectively. The sheep were sacrificed by bleeding of the carotid artery for two minutes. 17 tissues were immediately snap-frozen in liquid nitrogen for total RNA extraction. RNA extraction was performed using TRIzol reagent (TaKaRa, Dalian, China). TURBO DNA-free Kit (Ambion, Austin, TX, USA) was used for DNase treatment of total RNA. cDNA for gene amplification and expression was synthesized using PrimeScriptTMRT reagent kit (TaKaRa, Dalian, China). An equal volume of cDNA sample from the same tissue of each of six individuals was pooled for RT-PCR. The reactions were followed according the method described previously [12].

Next, real-time PCR was used to quantify expression levels of *BMP2*, *HAO1* and internal control *β-actin* in tissue samples described above. Additional samples of adipose and uterine tissues (*n* = 8 per tissue type and tail type) were used for validation of the differential expression of *BMP2* gene between tail types. For each measurement, real-time PCR was performed three times and the average gene expression was calculated. Amplification was followed as described in a previous study [12]. Real-time PCR results were processed by the 2^-ΔΔCt^ method [51]. The expression of *BMP2* in the perirenal adipose tissues of TBS was defined as 1.0 to calculate relative expression levels of four genes.

### Neutral test for retro-gene

Coding sequences of *PPP1CC* genes (sheep, goat, cattle, human, mouse and clawed frog), as well as retro-*PPP1CC* genes (sheep and goat) were aligned using MUSCLE algorithm [52] to generate phylogenetic tree. The topology (as showed in Fig. 3b) and codon alignment was treated as the input into PAML [53] “codeml” program to perform a log-ratio test (LRT). In this test, we defined part of the tree as a foreground branch (two r-*PPP1CC* sequences), and the rest as the background branch (all *PPP1CC* sequences). dN/dS ratio *ω* was calculated in these branches separately. To test whether *ω*_foreground_ equaled 1 (which means the evolution of r-*PPP1CC* putative codons is not different from non-functional sites), we calculated the log-scaled likelihood (*lnL*) under model M0 and M1. Under the “null model” M0, *ω*_foreground_ was pre-defined as *ω*_foreground_ = 1; while in M1, *ω*_foreground_ was calculated based on observed substitution rates. Two-fold difference of *lnL* between these two models (*2dlnL*) and *P*-value of LRT were then calculated as a statistical measurement (Additional file 1: Table S6).

## Additional files

Additional file 1: **Table S1.** List of 30-kb windows on MGS genome with top 0.5% high-LSBL values. **Table S2.** Information of four genotyped SNPs in association study. **Table S3.** Genotypes and phenotypes of 116 crossbreeding sheep. **Table S4.** Linear regression models for genotype-to-phenotype association test. **Table S5.** Primer sequences in the present study. **Table 6.** Neutral test for retro-*PPP1CC* gene. (XLS 162 kb)

Additional file 2: **Figure S1.** Evidence of selective sweep at chromosome 15 near *PDGFD*. Plots of selective sweep statistics at chromosome 15, from top to bottom: (1) *LSBL*; (2) pair-wise genetic distance *d*_xy_; (3) intra-lineage heterozygosity *H*_P_ (standardized). **Figure S2.** Validation of LOC101117953 DNA sequence. (**A**) Electrophoresis of PCR product captured by LOC101117953-specific primers. (**B**) Comparison between the result of PCR product sequencing and the reference assembly of sheep (oviAri3). **Figure S3.** Dot-plots for alignment between eight gene sequences and the sheep genome at chromosome 13. Alignments generated by LASTZ were visualized as dot plots using R script. Exon positions on the normal paralog are showed in blue boxes. **Figure S4.** Features of different tail types in the hybrid population. Pictures taken for two individual sheep from the hybrid population with thin tail (left) and fat tail (right). **Figure S5.** Tissue-expression patterns of genes near IBH region. Primers were designed to capture cDNA of five genes from ovine transcriptome. M, marker; 1, pituitary; 2, hypothalamus; 3, cerebellum; 4, cerebrum; 5, ovary; 6, oviduct; 7, cornua uterus; 8, corpus uterus; 9, thyroid; 10, adrenal gland; 11, heart; 12, liver; 13, spleen; 14, lung; 15, kidney; 16, perirenal adipose; 17, tail adipose. **Figure S6.** Stop-gain mutation on the putative protein sequence of LOC101117953. The position and type of the mutation which truncates the putative protein encoded by LOC101117953 (or r-*PPP1CC*). The truncated codon is highlighted in red. **Figure S7.** Chromatin interactions detected in human ESC. Sheep IBH region is homologous to the displayed area on human chromosome 20, between BMP2 and HAO1. Interaction density is visualized by the 3D Genome Browser (http://promoter.bx.psu.edu/hi-c/), using H1-ESC data and a resolution of 10 K. (DOC 1839 kb)

## Abbreviations

BMP2: Bone morphogenetic protein 2
EUS: European sheep breeds
HOA1: Hydroxyacid oxidase (glycolate oxidase) 1
IBH region: Intergenic region between BMP2 and HAO1
IROA: Iran sheep
LINE: Long interspersed nuclear element
LSBL: Lineage-specific branch length
LTR: Long terminal repeat
MGS: Chinese Mongolian sheep breeds
MOOA: Northern Africa sheep
PDGFD: Platelet-derived growth factor D
PPP1CC: Protein phosphatase 1 catalytic subunit gamma
qPCR: Quantitative PCR
r-PPP1CC: Retro-copy of PPP1CC
RTE: Retro-transposable elements
TBS: Chinese Tibetan sheep breeds
