# Supplementary material for: Rapid evolution of a retro-transposable hotspot of ovine genome underlies the alteration of BMP2 expression and development of fat tails
Source: BMC Genomics. 2019 Apr 2;20:261. doi: 10.1186/s12864-019-5620-6 (PMC6445056; doi:10.1186/s12864-019-5620-6)
Supplement: Supplementary file 2 — Figure S1. Evidence of selective sweep at chromosome 15 near PDGFD. Plots of selective sweep statistics at chromosome 15, from top to bottom: (1) LSBL; (2) pair-wise genetic distance dxy; (3) intra-lineage heterozygosity HP (standardized). Figure S2. Validation of LOC101117953 DNA sequence. (A) Electrophoresis of PCR product captured by LOC101117953-specific primers. (B) Comparison between the result of PCR product sequencing and the reference assembly of sheep (oviAri3). Figure S3. Dot-plots for alignment between eight gene sequences and the sheep genome at chromosome 13. Alignments generated by LASTZ were visualized as dot plots using R script. Exon positions on the normal paralog are showed in blue boxes. Figure S4. Features of different tail types in the hybrid population. Pictures taken for two individual sheep from the hybrid population with thin tail (left) and fat tail (right). Figure S5. Tissue-expression patterns of genes near IBH region. Primers were designed to capture cDNA of five genes from ovine transcriptome. M, marker; 1, pituitary; 2, hypothalamus; 3, cerebellum; 4, cerebrum; 5, ovary; 6, oviduct; 7, cornua uterus; 8, corpus uterus; 9, thyroid; 10, adrenal gland; 11, heart; 12, liver; 13, spleen; 14, lung; 15, kidney; 16, perirenal adipose; 17, tail adipose. Figure S6. Stop-gain mutation on the putative protein sequence of LOC101117953. The position and type of the mutation which truncates the putative protein encoded by LOC101117953 (or r-PPP1CC). The truncated codon is highlighted in red. Figure S7. Chromatin interactions detected in human ESC. Sheep IBH region is homologous to the displayed area on human chromosome 20, between BMP2 and HAO1. Interaction density is visualized by the 3D Genome Browser (http://promoter.bx.psu.edu/hi-c/), using H1-ESC data and a resolution of 10 K. (DOC 1839 kb) [file 12864_2019_5620_MOESM2_ESM.doc]

**Supplementary Materials for**

**Rapid evolution of a retro-transposable hotspot of ovine genome underlies the alteration of BMP2 expression and development of fat tails**

Zhangyuan Pan^†,1,3^, Shengdi Li^†,2,4^, Qiuyue Liu^1^, Zhen Wang^2^, Zhengkui Zhou^1^, Ran Di^1^, Xuejiao An^1^, Benpeng Miao^2,4^ , Xiangyu Wang^1^, Wenping Hu^1^, Xiaofei Guo^1^, Shenjin Lv^3^, Fukuan Li^3^, Guohui Ding^2,4^, Mingxing Chu*^,1^ & Yixue Li*^,2,4^

^1^Institute of Animal Science, Chinese Academy of Agricultural Sciences, Beijing, China.

^2^Key Lab of Computational Biology, CAS-MPG Partner Institute for Computational Biology, Shanghai Institutes for Biological Sciences, Chinese Academy of Sciences, Shanghai, China.

^3^College of Agriculture and Forestry Science, Linyi University, Linyi, China

^4^Shanghai Center for Bioinformation Technology, Shanghai Industrial Technology Institute, Shanghai, China.

^†^These authors contributed equally to this work.

*These authors jointly supervised this work.

Correspondence should be addressed to Y.L. (yxli@sibs.ac.cn) or M.C. (mxchu@263.net)

**Supplementary Figures**

**
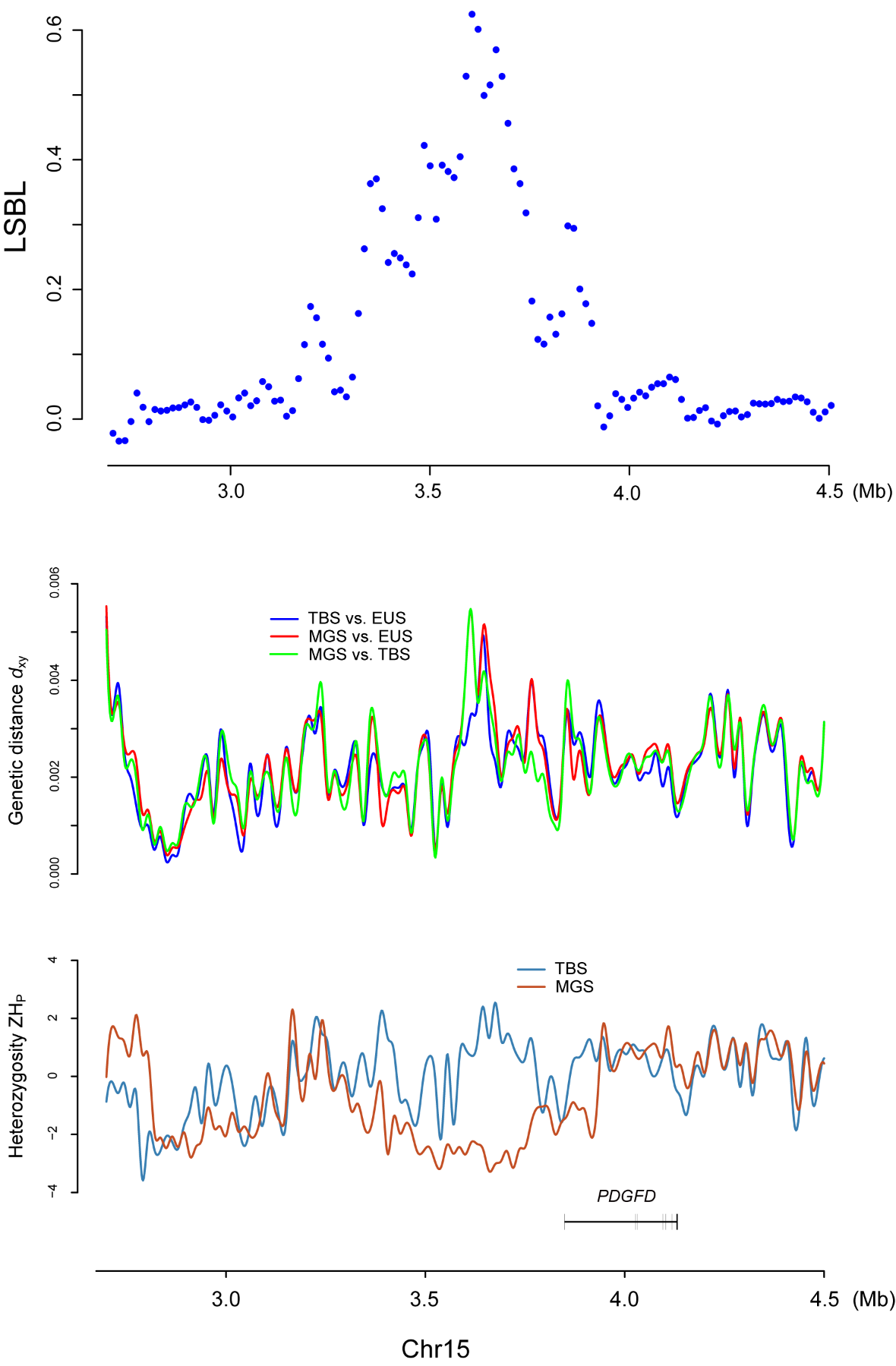
**

**Figure S1. Evidence of selective sweep at chromosome 15 near *PDGFD*.** Plots of selective sweep statistics at chromosome 15, from top to bottom: (1) *LSBL*; (2) pair-wise genetic distance *d*_xy_; (3) intra-lineage heterozygosity *H*_P_ (standardized).


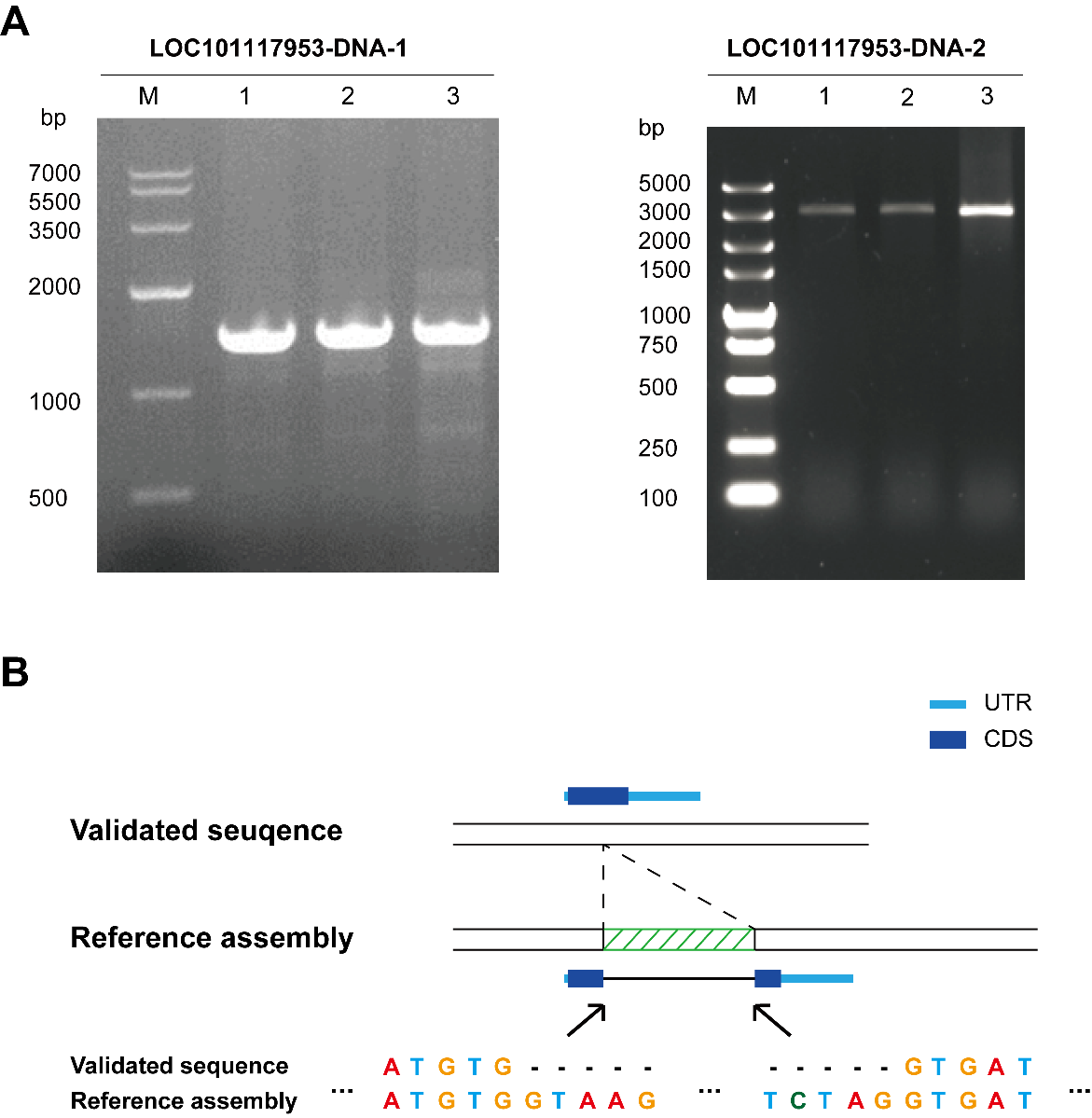


**Figure S2. Validation of LOC101117953 DNA sequence.** (**A**) Electrophoresis of PCR product captured by LOC101117953-specific primers. (**B**) Comparison between the result of PCR product sequencing and the reference assembly of sheep (oviAri3).

**
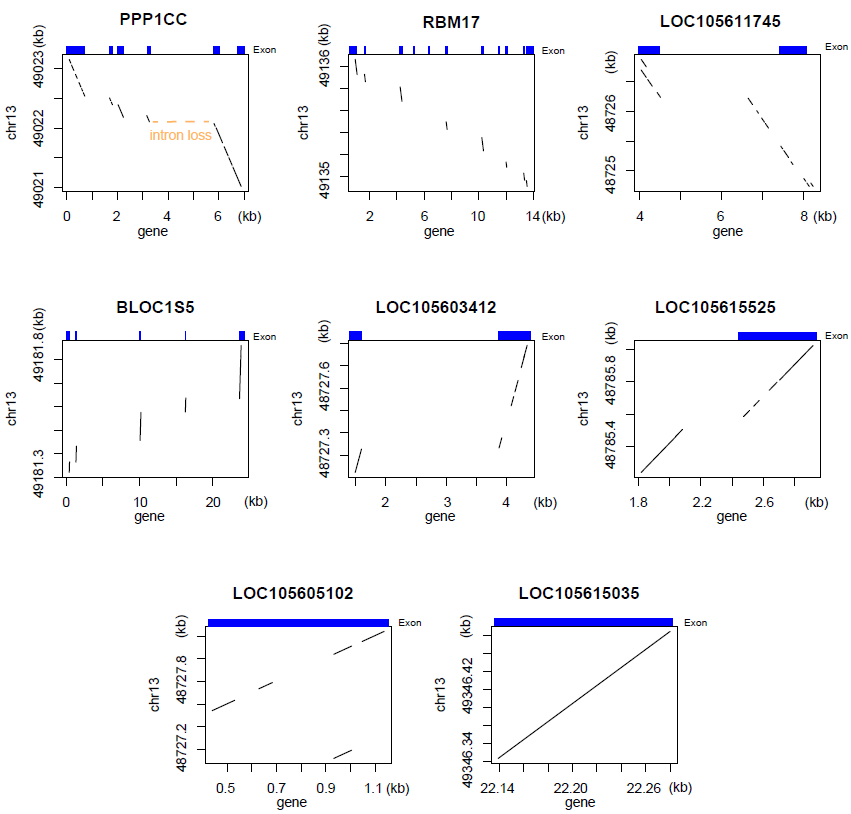
**

**Figure S3. Dot-plots for alignment between eight gene sequences and the sheep genome at chromosome 13.** Alignments generated by LASTZ were visualized as dot plots using R script. Exon positions on the normal paralog are showed in blue boxes.

**
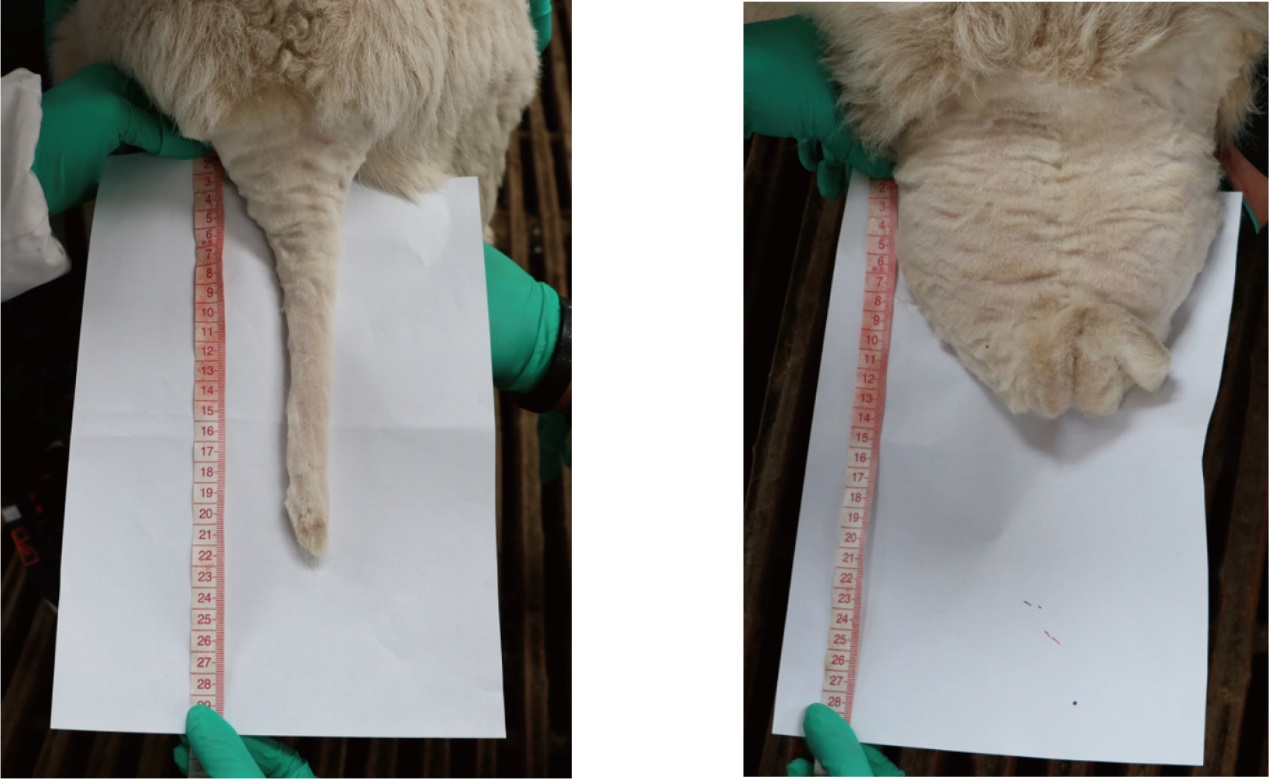
**

**Figure S4. Features of different tail types in the hybrid population.** Pictures taken for two individual sheep from the hybrid population with thin tail (left) and fat tail (right).

**
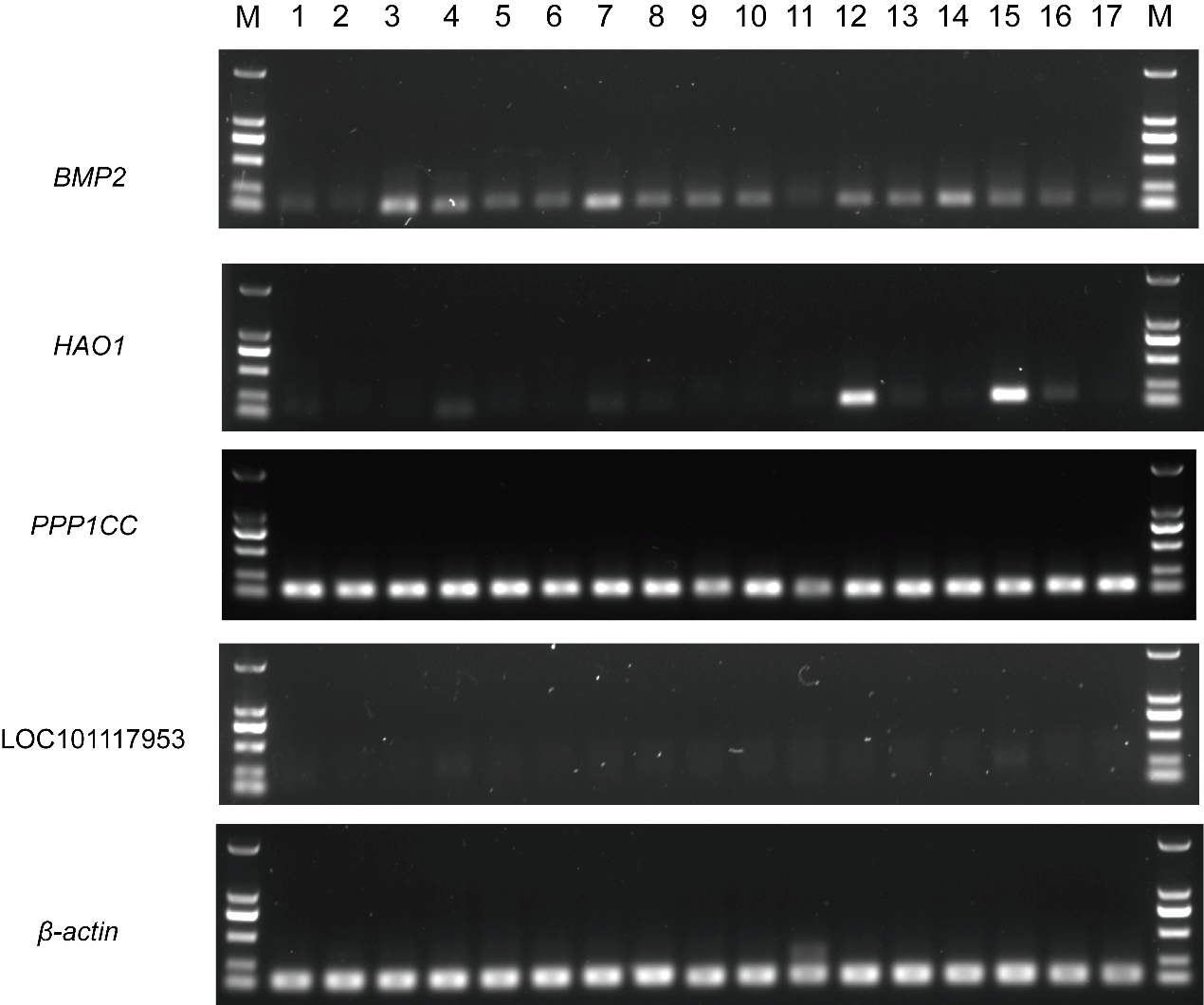
**

**Figure S5. Tissue-expression patterns of genes near IBH region.** Primers were designed to capture cDNA of five genes from ovine transcriptome. M, marker; 1, pituitary; 2, hypothalamus; 3, cerebellum; 4, cerebrum; 5, ovary; 6, oviduct; 7, cornua uterus; 8, corpus uterus; 9, thyroid; 10, adrenal gland; 11, heart; 12, liver; 13, spleen; 14, lung; 15, kidney; 16, perirenal adipose; 17, tail adipose.

**
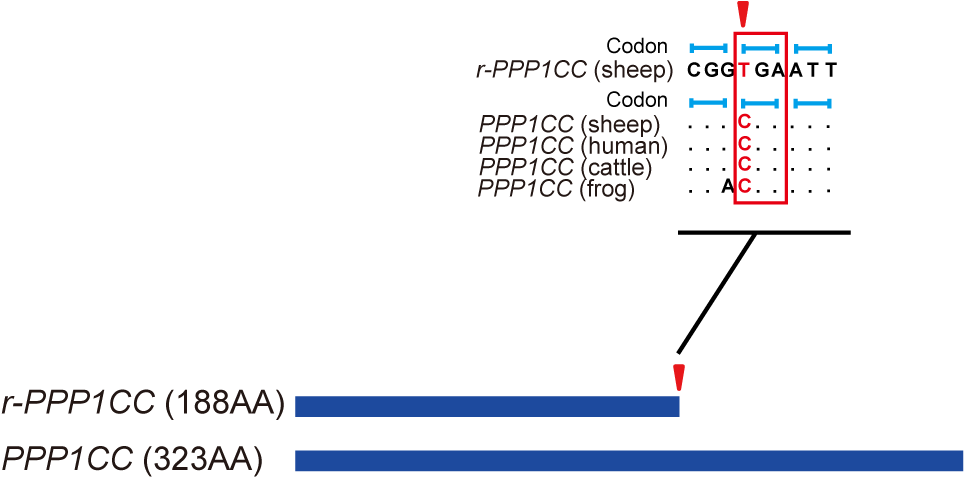
**

**Figure S6. Stop-gain mutation on the putative protein sequence of LOC101117953.** The position and type of the mutation which truncates the putative protein encoded by LOC101117953 (or r-*PPP1CC*). The truncated codon is highlighted in red.


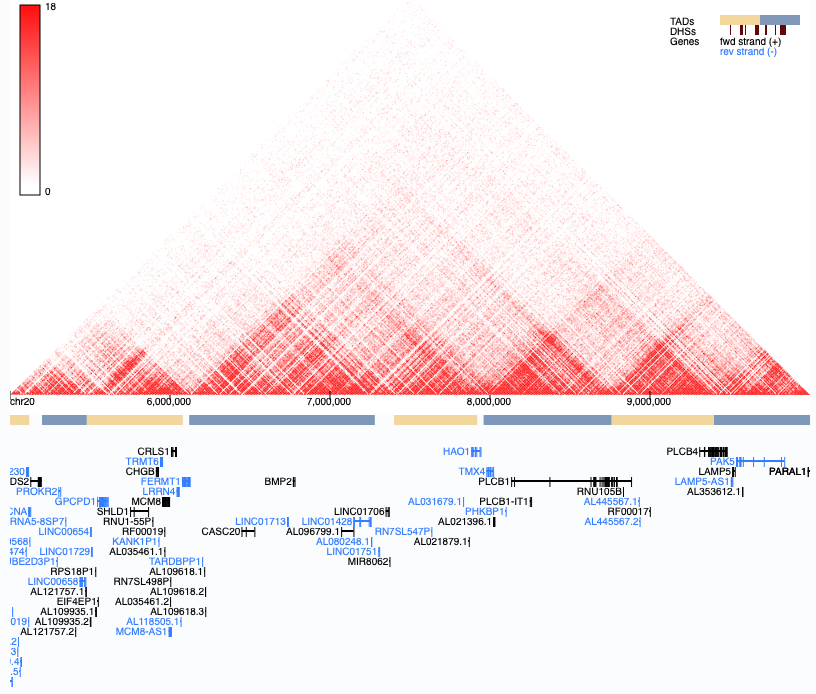

**Figure S7.** Chromatin interactions detected in human ESC. Sheep IBH region is homologous to the displayed area on human chromosome 20, between BMP2 and HAO1. Interaction density is visualized by the 3D Genome Browser (<http://promoter.bx.psu.edu/hi-c/>), using H1-ESC data and a resolution of 10K.
